# Darbepoetin, effective treatment of anaemia in paediatric patients with chronic renal failure

**DOI:** 10.1007/s00467-006-0402-1

**Published:** 2007-05-01

**Authors:** Jean-Luc André, Georges Deschênes, Bernard Boudailliez, Françoise Broux, Michel Fischbach, Marie-France Gagnadoux, Benjamin Horen, Annie Lahoche-Manucci, Marie-Alice Macher, Bernard Roussel, Michel Tsimaratos, Chantal Loirat

**Affiliations:** 1 Pediatric Nephrology Unit, Hôpital d’Enfants, CHU de Nancy, Rue du Morvan, 54511 Vandoeuvre les Nancy, France; 2 Pediatric Nephrology Unit, Assistance Publique-Hôpitaux de Paris, Hôpital Trousseau, 75012 Paris, France; 3 Pediatric 1, Hôpital Nord, CHU, 80030 Amiens, France; 4 Pediatric Nephrology Unit-CHU, Hopital Charles Nicolle, 76031 Rouen, France; 5 Pediatric 1, Hopital de Hautepierre-CHU, 67098 Strasbourg, France; 6 Pediatric Nephrology Unit, Assistance Publique-Hôpitaux de Paris, Hôpital Necker-Enfants Malades, 75743 Paris, France; 7 Pediatric Nephrology Unit, Hópital des Enfants-CHU, 31026 Toulouse, France; 8 Pediatric Nephrology Unit, Hópital Jeanne de Flandre-CHU, 59037 Lille, France; 9 Pediatric Nephrology Unit, Assistance Publique-Hôpitaux de Paris, Hôpital Robert Debré, 75019 Paris, France; 10 Pediatric Nephrology Unit, American Memorial Hospital-CHU, 51092 Reims, France; 11 Pediatric Nephrology Unit, Assistance Publique-Hôpitaux de Marseille, Hôpital de la Timone-Enfants, Marseille, France

**Keywords:** Darbepoetin alfa, Paediatrics, Chronic kidney disease, Anaemia, r-HuEPO, Dialysis, Haemoglobin, Chronic renal failure

## Abstract

Darbepoetin alfa (DA) is a unique long-acting treatment for anaemia in patients with chronic renal failure (CRF). This study assessed the mean dose of DA to achieve and maintain haemoglobin (Hb) levels between 11 g/dl and 13 g/dl in CRF children aged 11 years to 18 years. This observational, prospective study was conducted in 39 patients treated with DA. Twenty-nine patients were switched from recombinant human erythropoietin (r-HuEPO), and ten patients were naive to r-HuEPO. Naive patients received initial doses of 0.45 μg/kg of DA. Switched patients received a dose adjusted to the prior dose of r-HuEPO (200 IU r-HuEPO:1 μg DA). Among the switched patients, 79.3% received dialysis. No naive patients underwent dialysis. Overall, 74% of patients showed increased Hb level, with a mean value of 11.6 ± 1.6 g/dl, using a mean DA dose of 0.63 ± 0.48 μg/kg per week, and 66.7% patients reached the target Hb level. Hb increased in naive patients from 9.5 (95% CI: 7.7, 11.4) to 11.7 (95% CI: 10.9, 12.6) g/dl and in switched patients from 11.1 (95% CI: 10.6, 11.5) to 11.5 (95% CI: 10.8, 12.2) g/dl). Higher doses of DA were needed in the “switched” than in the “naive” patients to maintain Hb levels over 11 g/dl, respectively 0.73 (95% CI: 0.54, 0.92) and 0.34 (95% CI: 0.16, 0.52) μg/kg per week. Our results indicate the doses of DA necessary to treat CRF patients aged 11 years to 18 years. DA was an effective treatment to stabilise CRF patients at extended dosing intervals.

## Introduction

The dysfunction of erythropoietin (EPO) production because of declining renal function is the major cause of anaemia in patients with chronic renal failure. Therefore, dialysis patients with anaemia have an increased risk for cardiovascular morbidity and mortality [1]. Correction of anaemia through erythropoiesis stimulation leads to decreased blood transfusion requirements and anti-HLA immunisation [2], increased appetite and tolerance to exercise [3, 4] and improved school performance or quality of life [5, 6]. Recombinant human erythropoietin (r-HuEPO) has become the standard of care in treating anaemia associated with chronic renal failure (CRF) in adults [7–9] as well as in children [10, 11]. Conventional r-HuEPO requires two to three subcutaneous or intravenous injections per week over long periods of time, until successful kidney transplantation restores kidney function. By contrast, darbepoetin alpha (DA) that has an increased sialic acid carbohydrate content promotes decreased clearance and has a longer serum half-life than r-HuEPO [12–14], allowing a single weekly administration. Previous studies showed a similar potent effect of darbepoetin in adult as well as in paediatric patients either in pre-dialysis chronic renal failure or in end stage renal failure [8, 14–19]. The present study aims to determine the mean dose of darbepoetin necessary to achieve and maintain haemoglobin levels between 11.0 g/dl and 13.0 g/dl in CRF patients aged from 11 years to 18 years.

## Subjects and methods

### Patients

This study was composed of 54 children aged between 11 years and 18 years with CRF, in stable condition, either needing dialysis (haemodialysis or peritoneal dialysis) or not, either pretreated or not with r-HuEPO and having transferrin saturation (T-sat) ≥20%. and/or serum ferritin ≥50 μg/l [the Kidney Disease Outcomes Quality Initiative (KDOQI) had not yet been published at the protocol onset, but results were analysed according to serum ferritin concentrations greater or less than 100 μg/l]. The patients were classified as “switched” patients (previously treated by r-HuEPO), or “naive” (never treated with r-HuEPO). Written informed consent was obtained from all patients and/or their parents.

Patients with uncontrolled hypertension, cardiac failure, malignancy and/or haematological disease, known resistance to r-HuEPO, or recent (within 3 months) kidney transplantation were excluded.

### Methodology

The study was observational and prospective. Data for each patient were obtained monthly for 6 months. Once included, the patients received a dosage of darbepoetin (Aranesp®) according to their previous treatment status and as recommended in the summary of product characteristics “Naive” patients received darbepoetin at initial doses of approximately 0.45 μg/kg once a week. However, as darbepoetin is supplied in pre-filled syringes containing from 10 μg to 60 μg (by steps of 10 μg), some patients received slightly lower or higher adjusted doses than 0.45 μg/kg, because dose accuracy cannot be guaranteed if the syringe is partially injected. In fact, doses were pulled out to the upper range in a majority of patients. The real dose received by each patient was recorded (Table 2).

For “switched” patients, the initial dose of darbepoetin was calculated from the prior dose of r-HuEPO according to the following conversion index: 1 μg darbepoetin for 200 IU r-HuEPO. Patients receiving r-HuEPO two to three times weekly were switched to once-weekly darbepoetin, while those receiving weekly r-HuEPO were switched to every-other-week darbepoetin.

### Statistical analysis

The population analysis considered all patients receiving the treatment and with Hb concentration evaluated at month 6 (M6). Those patients receiving a transplant during the study were not included in the analysis. Summary statistics (mean, median, SD, and number and percentage of subjects in each group for categorical variables) are presented for baseline and M6. Changes in Hb and darbepoetin dose comparing the baseline to M6 are summarised, with means, SD and two-sided 95% confidence intervals (95% CI).

Fisher’s exact test was used to compare the categorical variables, such as gender, dialysis, ferritin and T-sat between the study groups. Continuous data such as age at inclusion, weight, height, haemoglobin level and weighted doses of darbepoetin alfa were compared by the Wilcoxon–Mann-Whitney test, a nonparametric test for independent samples.

## Results

### Patient disposition

Among the 54 chronic renal failure paediatric patients enrolled at 12 sites from January 2003 to April 2004, 15 were discarded during the study due to kidney transplantation (*n* = 14) and adverse event (one patient with abdominal pain). The final analysis included 39 patients (*n* = 10 in the “naive group”, and *n* = 29 in the “switched group”). Original diseases were renal hypoplasia–dysplasia with or without uropathy in eight, hereditary disease in nine, glomerulonephritis in ten, haemolytic uraemic syndrome in five, systemic disease in two, interstitial nephritis in one and unknown in four. At baseline, 22 patients received intravenous (i.v.) injections of darbepoetin and 17 patients received subcutaneous (s.c.) injections of darbepoetin. At M6, 25 patients received i.v. injections and 14 received s.c. injections.

### Demographics and baseline characteristics

The groups were comparable for gender and age, while weight and height were lower in the switched group (Table 1). No patients in the naive group underwent dialysis. By contrast, 23 patients (79.3%) in the switched group received dialysis (mainly haemodialysis, Table 1). This dialysis status could affect the results.

**Table 1:** Demographic and baseline characteristics (*n* = 39) (*Naive* not previously treated with r-HuEPO, *Switched* previously treated with r-HuEPO, *T-sat* transferrin saturation

Characteristics	Naïve (*n* = 10)	Switched (*n* = 29)	Total (*n* = 39)	*P* ^a^
Gender (male/female)	4/6	15/14	19/20	0.716
Age (years)^b^	15.7 ± 2.0	15.1 ± 2.2	15.2 ± 2.1	0.412
Weight (kg)^b^	49.5 ± 14.7	38.7 ± 7.8	41.4 ± 10.9	0.030
Height (cm)^b^	152.0 ± 12.6	148.0 ± 12.0	149.0 ± 12.1	0.281
Dialysis, *n* (%)^c^	0 (0.0)	23 (79.3)	23 (58.9)	<0.001
Haemodialysis	0 (0.0)	17 (73.9)	17 (73.9)	
Peritoneal dialysis	0 (0.0)	6 (26.1)	6 (26.1)	
Haemoglobin, *n*	9	29	38	
Mean ± SD (g/dl)	9.5 ± 2.4	11.1 ± 1.2	10.7 ± 1.7	0.140
Hb<11 g/dl^c^	6 (66.7)	14 (48.3)	20 (52.6)	0.454
Ferritin, *n*	9	25	34	
Mean ± SD (μg/dl)	202.8 ± 190.2	391.2 ± 339.6	341.3 ± 315.8	0.154
<100 (μg/L), ^c^	5 (55.6)	6 (24.0)	11 (32.4)	0.111
T-sat, *n*	5	22	27	
Mean ± SD (μg/dl)	35.7 ± 12.9	32.5 ± 18.3	33.1 ± 17.2	0.492
<20%, *n* (%) ^c^	0 (0.0)	4 (18.2)	4 (14.8)	0.561

^a^Statistical tests: Wilcoxon-Mann-Whitney exact test for continuous variables and Fisher’s exact test for categorical variables

^b^Values expressed as mean ± standard deviation

^c^Number of patients/number of examined patients (%)

As expected, the Hb levels and iron status at baseline differed between the two groups. Mean Hb level was 9.5 ± 2.4 g/dl in the naive patients, while switched patients were stabilised at 11.1 ± 1.2 g/dl of Hb, due to their previous treatment.

### Dosage of darbepoetin

When M3 was compared with baseline, the Hb levels increased in 34 patients (87%), showing a mean increase of 1.8 g/dl (+17%). The doses of darbepoetin subsequently decreased from 0.73 μg/kg per week to 0.68 μg/kg per week (−7%). When M6 was compared with baseline (Table 2), the Hb levels increased in 29 patients (74%), reaching a mean increase of 0.8 g/dl (+8.4%), despite a dose adjustment at 0.63 μg/kg per week at M6.

**Table 2:** Hb levels, doses of darbepoetin alfa and iron status according to the previous treatment (*Naive* not previously treated with r-HuEPO, *Switched* previously treated with r-HuEPO)

Parameter	Naïve (*n* = 10)^a^	Switched (*n* = 29)	Total (*n* = 39)	*P* ^b^
Hb, g/dl				
Baseline				
Mean ± SD	9.5 ± 2.4	11.1 ± 1.2	10.7 ± 1.7	0.140
95% CI	[7.7; 11.4]	[10.6; 11.5]	[10.2; 11.3]	
M6				
Mean ± SD	11.7 ± 1.2	11.5 ± 1.7	11.6 ± 1.6	0.822
95% CI	[10.9; 12.6]	[10.8; 12.2]	[11.0; 12.1]	
Change^c^				
Mean ± SD	2.1 ± 2.6	0.4 ± 2.1	0.8 ± 2.3	0.126
95% CI	[0.1; 4.1]	[−0.4; 1.2]	[0.1; 1.6]	
Doses, μg/kg per week				
Baseline				
Mean ± SD	0.53 ± 0.23	0.80 ± 0.55	0.73 ± 0.50	0.159
95% CI	[0.35; 0.70]	[0.59; 1.01]	[0.57; 0.90]	
M6				
Mean ± SD	0.34 ± 0.25	0.73 ± 0.50	0.63 ± 0.48	0.015
95% CI	[0.16; 0.52]	[0.54; 0.92]	[0.48; 0.79]	
Change^c^	−35.8%	−8.8%	−13.7%	
Ferritin <100 μg/l				
Baseline	5/9 (55.6)^d^	6/25 (24.0)^d^	11/34 (32.4)^d^	0.111
M6	2/9 (22.2)^d^	3/25 (12.0)^d^	5/34 (14.7)^d^	0.591
T-sat <20%				
Baseline	0/5 (0.0)^d^	4/22 (18.2)^d^	4/27 (14.8)^d^	0.561
M6	0/5 (0.0)^d^	5/21 (23.8)^d^	5/26 (19.2)^d^	0.545

^a^*n* = 9 for Hb levels at baseline

^b^Statistical tests: Wilcoxon-Mann-Whitney exact test for continuous variables and Fisher’s exact test for categorical variables

^c^Change values were calculated comparing the baseline to M6

^d^Number of patients/number of examined patients (%)

**Fig. 1:**
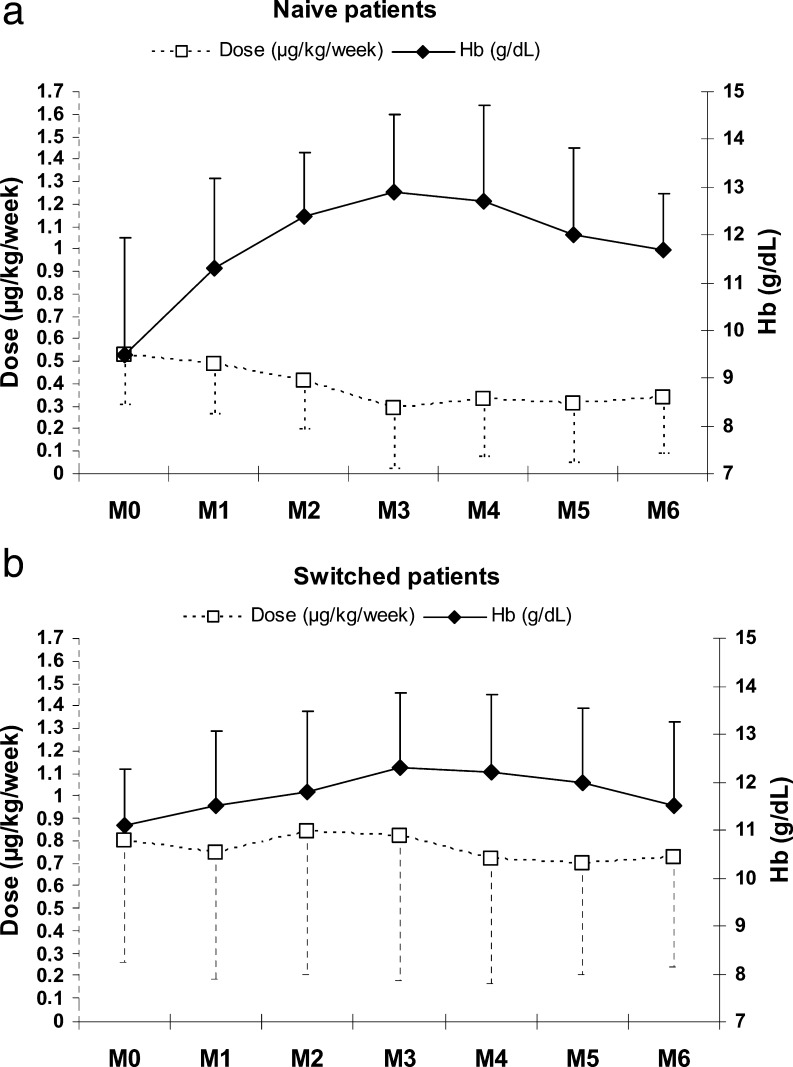
Evolution of haemoglobin (Hb) concentration (g/dl) and darbepoetin alfa dose (μg/kg per week) over time according to previous treatment. Naive patients, *n* = 10 (**a**) and switched patients, *n* = 29 (**b**). (Mean and SD)

The naive and switched groups displayed different behaviours: in the naive patients, the mean levels of Hb increased by 3.2 g/dl up to M3, then decrease by 1 g/dl between M3 and M6, while darbepoetin doses were stable during this period (Fig. 1a). Thus, the levels of Hb had increased to 11.7 ± 1.2 g/dl (+2.1 g/dl or +22%) by M6, compared with those at baseline (Table 2) with a final dose of 0.34 ± 0.25 μg/kg per week (Fig. 1a), representing a 36% reduction compared to the initial doses. In the “switched” patients the mean Hb had increased to 12.3 ± 1.5 g/dl by M3 and had stabilised at 11.5 ± 1.7 g/dl (+0.4 g/dl or +4%) by M6, while the final dose of 0.73 (±0.50) μg/kg per week was quite similar to the initial doses (Fig. 1b). Finally, in the period M3–M6, darbepoetin doses were higher in the switched patients than in the naive patients for similar Hb levels (Table 2).

### Evolution of DA doses and Hb

Among the 39 patients, 26 patients (66.7%) reached a target Hb >11 g/dl. In these 26 patients who maintained the target Hb at M6 (Hb ≥11 g/dl group), the mean Hb level increased by 1.7 g/dl, with a concomitant reduction of 24% in the mean darbepoetin dose (Table 3). Among the Hb ≥ 11 g/dl group (*n* = 26), 19 patients belonged to the switched group and seven to the naive group. By contrast, in the Hb<11 g/dl group (the 13 remaining patients) the mean Hb level decreased by 0.89 g/dl, in spite of an increase of 7% of the mean darbepoetin doses. Among these 13 patients, a mean Hb increase of 3.5 g/dl was observed in six patients, but this increase of Hb level failed to reach the target because the initial Hb levels were very low. Among the seven patients with erythropoietin resistance during the 6-month follow-up, one had a primary infection of tuberculosis, one underwent orthopaedic surgery, two stopped the treatment for 1 month, one had an inappropriate dosage of darbepoetin and two had an unexplained decrease in Hb level between M5 and M6).

**Table 3:** Hb levels, darbepoetin doses and iron status according to the target Hb at M6 (*Naive* not previously treated with r-HuEPO, *Switched* previously treated with r-HuEPO)

Parameter	Hb ≥11 g/dl (*n* = 26)	Hb <11 g/dl (*n* = 13)	*P* ^a^
Hb, g/dl			
Baseline			
Mean ± SD	10.8 ± 1.5	10.6 ± 2.0	0.853
95% CI	[10.2; 11.4]	[9.3; 11.8]	
M6			
Mean ± SD	12.5 ± 0.9	9.8 ± 1.0	<0.001
95% CI	[12.1; 12.8]	[9.2; 10.4]	
Change^b^			
Mean ± SD	1.7 ± 1.8	−0.8 ± 2.4	0.001
95% CI	[0.9; 2.4]	[−2.3; 0.6]	
Doses (μg/kg per week)			
Baseline			
Mean ± SD	0.75 ± 0.48	0.71 ± 0.54	0.580
95% CI	[0.55; 0.95]	[0.38; 1.04]	
M6			
Mean ± SD	0.57 ± 0.37	0.76 ± 0.64	0.447
95% CI	[0.42; 0.72]	[0.37; 1.14]	
Change^b^	−24.0%	+7.0%	
Ferritin <100 μg/l			
Baseline	6/23 (26.1)^c^	5/11 (45.5)^c^	0.434
M6	4/21 (19.1)^c^	1/13 (7.7)^c^	0.627
T-sat <20%			
Baseline	2/19 (10.5)^c^	2/8 (25.0)^c^	0.558
M6	2/16 (12.5)^c^	3/10 (30.0)^c^	0.340

^a^Statistical tests: Wilcoxon-Mann-Whitney exact test for continuous variables and Fisher’s exact test for categorical variables

^b^Change values were calculated comparing the baseline to M6

^c^Number of patients/number of examined patients (%)

### Iron supplementation, ferritin and T-sat levels over the study

The proportion of patients with ferritin <100 μg/l decreased from baseline to M6, from 55.6% to 22.2% and from 24% to 12% for naive patients and switched patients, respectively (Table 2). Many patients had changes in their mode of administration, either i.v. or oral supplementation during the 6 months of the study. At baseline, 14 patients received oral supplementation (seven in the naive group), and 17 received i.v. supplementation (one naive). No transferrin values <20% were seen for naive patients over the follow-up, while the proportion of switched patients having transferrin values <20% increased by 5.6% (Table 2). Interestingly, the proportion of patients with ferritin <100 μg/l at baseline was higher in the group who had not achieve the target Hb>11 g/dl (Table 3).

### Dosing intervals

At baseline, 92.1% patients—all of the naive group and 89.7% of the switched group—received darbepoetin once a week, and 7.9% received it every other week. At M6, 76.9% patients (30% naive and 93% switched), were still receiving darbepoetin once a week, 15.4% patients (40% naïve and 6.9% switched) received darbepoetin every other week, and 7.7% patients (30% naive patients and 0% switched) had one dose per month.

### Safety and tolerance

Two patients reported adverse events: vascular access thrombosis in one (Hb = 11.1 g/dl), and abdominal pain in a second, leading to their withdrawal from the study, although no relation with treatment was demonstrated in these two patients. One patient needed a transfusion at baseline (Hb 5.3 g/dl) and his inclusion is questionable.

## Discussion

The objective of this study was to determine the mean dose of darbepoetin necessary to control anaemia in CRF children aged 11 years to 18 years. The patients were followed-up for 6 months under usual routine practice. Two populations of patients were defined: “naive” patients (not previously treated with r-HuEPO) and “switched” patients (previously treated with r-HuEPO and switched to darbepoetin). The two populations differed by: (1) their baseline Hb levels, (2) their darbepoetin doses at inclusion, (3) dialysis status (none on dialysis in the naive group, 73.9% on dialysis—mainly haemodialysis—in the switched group). Our series showed that (1) darbepoetin efficacy was demonstrated in either naive patients or in switched patients, where Hb levels increased following 3 months of darbepoetin therapy, (2) darbepoetin doses were lowered by 36% to stabilise the haemoglobin level within the expected target in the naive patients and (3) darbepoetin dosage was twice as high in switched patients than in naive patients to maintain a similar haemoglobin level by 6 months of treatment.

These results extend those of recent publications where darbepoetin was found to be effective in controlling anaemia in seven children on haemodialysis [17] and in a group of 26 children across the spectrum of chronic renal failure to end-stage renal disease [18]. A decrease in the doses, together with less frequent injections, reinforces the interest of treating CRF patients with darbepoetin, owing to a parallel reduction in morbidity and care costs. Previous studies have shown that darbepoetin can effectively maintain haemoglobin concentrations when given at extended dose intervals relative to r-HuEPO in dialysis subjects [15, 19–21]. The conversion index of 1 μg darbepoetin/200 U r-HuEPO appeared satisfactory in our population, while maintaining stable Hb concentrations in the switched group.

In the present study, which included children aged 11 years to 18 years, a different behaviour was observed according to the previous treatment status, i.e. between the naive and the switched patients. Higher darbepoetin doses were needed in the switched patients to maintain the target Hb level. This difference was seen from the start (initial darbepoetin doses were proportional to the previous r-HuEPO dose) to the end of the study. Several factors could explain the differences in darbepoetin dose between the groups. Actually, 79% of patients of the switched group received dialysis, while no dialysis patient was included in the naive group. Thus, the stage of chronic kidney disease was more severe in the former group, with a possibly more reduced residual EPO secretion, more important blood loss and more accumulation of anti-erythropoiesis inhibitors [22]. In progressive renal failure, the rHuEPO dose required to attain target Hb levels was shown to increase as patients’ renal function deteriorated [23]. In addition, none of the naive patients versus 5/21 of switched patients had T-sat <20% at M6. An important consideration when one is treating anaemia in CRF patient is iron availability for erythropoiesis [24]. Despite the prescription of iron supplementation, the proportion of children with transferrin-saturation <20% appeared more important in the groups of patients having Hb <11 g/dl by M6. An adequate and continuous correction of the absolute and functional deficiency of iron reserves is necessary during treatment to optimise the darbepoetin efficacy in those patients, as recommended in KDOQI [25].

The tolerance of darbepoetin was excellent. In the present study one patient discontinued the treatment because of abdominal pain possibly related to darbepoetin. No injection pain was spontaneously reported during the patient’s follow-up. However, no specific question was included in the protocol.

In summary, darbepoetin alfa is a tool adapted to treat anaemia in children with chronic renal failure aged 11 years to 18 years at extended dosing intervals. Patients previously treated with r-HuEPO require higher darbepoetin doses than naive patients do, most likely due to a longer period of chronic renal failure, dialysis status, and reduced level of residual erythropoietin secretion, exacerbated iron loss and accumulation of erythropoiesis inhibitors. The clear decrease in dose and lengthening of the treatment interval in nearly all the naive patients (all pre-dialysis) suggest that in children with chronic renal disease—particularly if they do not yet require dialysis—the administration of darbepoetin alfa every 15 days from the start of the treatment may be a good option, as seems to be demonstrated in recent publications in adults [26].
